# The relationship between futile care perception and moral distress among intensive care unit nurses

**Published:** 2018-03-07

**Authors:** Hamid Asayesh, Mojtaba Mosavi, Mohammad Abdi, Mohammad Parvaresh Masoud, Kurosh Jodaki

**Affiliations:** 1 *Mentor, Spiritual Health Research Center, Department of Medical Emergencies, School of Paramedicine, Qom University of Medical Sciences, Qom, Iran.*; 2 *Assistant Professor, Department of Anesthesia, School of Paramedicine, Qom University of Medical Sciences, Qom, Iran.*; 3 *Mentor, Department of Intensive Care, School of Nursing and Midwifery, Zanjan University of Medical Sciences, Zanjan, Iran.*; 4 *Mentor, PhD Candidate, Department of Medical Emergencies, School of Paramedicine, Qom University of Medical Sciences, Qom, Iran.*; 5 *Mentor, Spiritual Health Research Center, Departments of Anesthesia, School of Paramedicine, Qom University of Medical Sciences, Qom, Iran.*

**Keywords:** *Futile care*, *Moral distress*, *Intensive Care Unit*

## Abstract

Moral distress is among the various types of distress that involves nurses and can lead to multiple complications. It is therefore rather important to identify the factors related to moral distress. The purpose of this study was to examine the relationship between futile care perception and moral distress among intensive care unit (ICU) nurses. This cross-sectional study used a descriptive-correlation method and was conducted on 117 ICU nurses of Qom hospitals in 2016. Data were collected using a 17-item futile care perception questionnaire, and Jameton’s moral distress questionnaire containing 30 questions. Data analysis was performed using SPSS 16, descriptive statistics and univariate regression analysis. The results showed that the mean age of the participants was 34.99, and most (about 66.7%) were women. Univariate regression analysis indicated that when ICU nurses’ perception of futile care and work experience increased, their moral distress also increased significantly (*P* = 0.03 and *P* = 0.02, respectively). It can therefore be concluded that moral distress is associated with futile care and ICU work experience. It seems that some interventions are necessary in future to place nurses in clinical situations involving futile care, and thus reduce their level of moral distress.

## Introduction

In nursing practice, burnout may be the result of various forms of distress. Moral issues are among the major sources of distress in clinical situations and therefore need to be adequately addressed (1, 2). Moral distress is a kind of pain or anger that affects the body, mind and communication, and is created in response to situations where the individual is aware of the existence of a moral problem (1). Jameton believes that moral distress is caused by several factors, and can lead to complications such as anger, disappointment, anxiety, sadness, headaches, frustration, depression and moral mistakes (2). Studies have shown that nurses may experience moral distress from once or twice a year to as often as once a week (3, 4). The results of various studies have indicated high (5), moderate (6, 7), or low (8) levels of moral distress among nurses. This type of distress can have many physical and psychological effects on nurses and influence their professional roles (9). Therefore, it is important to recognize the effective factors through considering the frequency of moral distress and its complications among ICU nurses (6). In her research, Corley concluded that moral distress occurs in a variety of medical situations, including providing end-of-life care for patients experiencing prolonged deaths and caring for those less likely to survive, as a response to their families’ request (10). On the other hand, the aim of nursing is to care for all patients regardless of their conditions, even when chances of life and recovery are low (11). This view can put nurses at risk because they may consider certain therapeutic measures as futile, and the coercion of nurses to carry out these measures may have complications for them due to moral contradictions (12). The definition of futile care varies according to the patients’ circumstances as well as the individual values of nurses (13). Futile care may be defined based on chances of survival or the quality of life afterwards (14). However, different perceptions of futile care as aggressive treatments or end-of-life care interventions pertain to patients whose life expectancies or chances of recovery are very low (15). According to this type of definition, if the therapeutic goals are not achievable or the success rate is too low, certain medical actions are considered ineffective (16), and will only impose additional costs on the medical system (17). One cause of moral distress among the medical team may be the various clinical situations that arise due to the advanced medical techniques; another cause is the increase in the number of elderly people, who are more likely to be exposed to futile care before they pass away (12). In such cases, the pain and discomfort of patients is untenable (1), and will give rise to moral distress in medical staff. Several studies have investigated moral distress among Iranian nurses and often have reported a considerable level among participants (18, 19). Moreover, a quantitative study in ICU has shown that futile action is one of the sources of moral distress (20). Most studies have found a significant relationship between moral distress and futile care perception (15, 21, 22), but findings pertaining to the relationship between these issues and the demographic variables of nurses are contradictory. Due to the nature of moral distress and futile care perception, and in order to better understand them in nursing care, they need to be investigated across the different regions of Iran. Therefore, the research team decided to examine the relationship between futile care perception and moral distress in ICU nurses of the hospitals affiliated to Qom University of Medical Sciences in Iran.

## Method

This was a descriptive, cross-sectional study and investigated the relationship between futile care perception and moral distress among ICU nurses of the hospitals affiliated to Qom University of Medical Sciences (Shahid Beheshti, Nekooie and Kamkar Hospitals) in 2016. Research population consisted of the ICU nurses of the above-mentioned hospitals, and samples were selected from among members of the research community who met the entry criteria and consented to participate in the study. The entry criteria included: 1) having a bachelor’s degree or higher in nursing, 2) having at least 6 months of ICU experience, and 3) full-time employment. Eventually, 117 full-time nurses participated in the study.

Data collection tool was a three-part questionnaire. The first part was a demographic questionnaire that included personal and professional characteristics such as age, sex, marital status, level of education, ICU experience, and experience of caring for patients at the end of life. The second part was a futile care perception questionnaire developed by Mohammadi and Roshanzadeh (16) and included 17 statements. Each statement presented a clinical situation that aimed to measure the nurses’ perceptions of futile care in terms of severity and frequency, designed based on Corley’s Moral Distress Scale. The questionnaire used a 6-point Likert scale arranged in severity from 0 (not at all) to 5 (very high), and in frequency from 0 (never) to 5 (repeatedly). Content validity and reliability of this questionnaire were reported by Mohammadi and Roshanzadeh to be 80% and 82%, respectively (16), and 82% and 85% respectively in a study by Borhani et al. (21). The third part was Jameton’s moral distress questionnaire that consists of 30 questions and has been verified by members of the Nursing Association of Iran in 2008 (23). This questionnaire comprises three sections: patient’s ignorance (16 questions), patient’s decision-making (8 questions), and professional performance competency of nurses (6 questions). The reliability of this questionnaire was determined through Cronbach's alpha test for patient's ignorance, patient’s decision-making and professional performance competency at 93%, 86% and 80%, respectively. After obtaining the legal and ethical licenses from Qom University of Medical Sciences, Iran, the questionnaires were sent to research departments, and the participants were provided with instructions on how to complete the questionnaires. It should be noted that the participants were informed about freedom of research participation. Data analysis was performed using SPSS 16, descriptive statistics and univariate regression tests.

## Results

Of the 130 critical care nurses who received the questionnaire, 117 nurses completed it (response rate = 90%). The average age of the study participants was 34.99, with a standard deviation of 7.34. The results showed that 66.7% of the study subjects were female and 33.3% were male, and 47% had more than four years of ICU experience (Table 1). The mean score of moral distress among the participants was found to be 137.53, with a standard deviation of 23.14. In addition, the mean scores of futile care perception in the two dimensions of severity and frequency were 74.45 ± 11.08 and 76.48 ± 13.57, respectively (Table 2). Univariate regression analysis demonstrated a significant relationship between futile care perception and moral distress in the two dimensions of severity (OR = 1.03, CI = 1.01 - 1.07) and frequency (OR = 1.03, CI = 1.00 - 1.06). Furthermore, the statistical analysis of the above-mentioned test showed a significant relationship between ICU experience (especially among the male participants) and moral distress (OR = 3.06, CI = 1.14 -8.19 and OR = 2.30, CI = 1.04 - 5.05) (Table 3).

**Table 1:** Demographic characteristics of the participants

**Variables**	**Mean ± Standard ** **Deviation**
**Age (Years)**	34.99 **± 7.54**
**Gender (number, %)** Male Female	39 (33.3) 78 (66.7)
**Work Experience in ICU ** **(Years) ** Less than 1 1 - 2 2 - 4 More than 4	17 **± 14.5** 18 **± 15.4** 27 **± 23.1** 55 **± 47.0**
**Educational Level** Bachelor’s degree Master’s degree	97 **± 82.9** 20 **± 17.1**
**Schedule** Permanent morning Permanent night Rotating day	12 **± 10.3** 9 **± 7.7** 96 **± 82.1**

**Table 2:** Mean and standard deviation of moral distress and futile care perception

**Variables**	**Mean ** **(SD)**	**Categories** **N (%)**		
		Low	Moderate	High
**Moral ** **Distress**	137.53 (23.14)	29 (24.8)	61 (52.1)	27 (23.1)
**Futile Care ** **(Severity)**	74.45 (11.08)	32 (27.4)	60 (51.3)	25 (21.4)
**Futile Care ** **(Frequency)**	76.48 (13.57)	32 (27.4)	58 (49.6)	27 (23.1)

**Table 3:** Univariate logistic regression results with moral distress as outcome variable

**Variables**	**OR**	**95% CI**	**P-Value**
**Age **	1.03	0.98 - 1.06	0.15
**Gender** Male Female	2.30 1	1.04 - 5.05 -	0.03 -
**Work Experience in ICU (Years) ** More than 4 2 - 4 1 - 2 Less than 1	3.06 0.82 0.70 1	1.14 - 8.19 0.27 - 2.43 0.22 - 2.17 -	0.02 0.72 0.54 1
**Educational Level** Bachelor’s degree Master’s degree	0.32 1	0.11 - 0.93 -	0.03 -
**Schedule** Permanent morning Permanent night Rotating day	0.75 0.82 1	0.46 - 2.27 0.40 - 1.97 -	0.95 0.92 -
**Futile Care (Severity)**	1.03	1.01 - 1.07	0.03
**Futile Care (Frequency)**	1.03	1.00 - 1.06	0.03

## Discussion

The results of this study showed that 75.2% of the ICU nurses who participated in this research had a moderate to high level of moral distress. In a study by Borhani et al. performed on Iranian ICU nurses, moderate levels of moral distress were reported (21). Findings of a hospital study conducted in Italy by Karanikola et al. were in accordance with the results of the present study as they reported a moderate to high level of moral distress among their ICU staff (24). In a study conducted by Lawrence in the United States during 2011, the level of moral distress among ICU nurses was also reported at a moderate to high level (25), which is similar to the results of the present study.

**ICU Experience and Moral Distress**

The results of this study showed a direct relationship between moral distress and ICU experience among nurses, that is, those with more than four years’ experience in ICU exhibited a significantly higher rate of moral distress. One explanation for this may be that working in the intensive care unit entails futile care, which is related to certain challenges and other sources of moral distress. These results are in accordance with the findings of a 2009 study by Epstein and Hamric (26), as well as one by Dodek et al. conducted in 2016 in Canada (27). These studies discovered a relationship between moral distress and work experience, that is, the longer the nurses worked in the ICU, the greater moral distress they would feel. In other words, having recurring experiences that cause moral distress leads to an exacerbation of primitive moral distress and the other anxieties that result from individuals’ previous experiences (26); in both cases, the level of moral distress will increase among nurses. Additionally, Mobley et al. believed increased moral distress among ICU nurses over time to be due to a lack of adaptation mechanisms for moral distress situations as well as frequent exposure to futile care situations (15). In another study, Elpern et al. found that moral distress is significantly associated with nursing experience (7). In their study of moral distress among Iranian nurses, Vaziri et al. noted that increased work experience significantly reduced job satisfaction among nurses, which affected their physical and mental health, self-image and spiritual life (18). They have believed these complications to be the outcome of intensified moral distress resulting from increased work experience. Borhani et al. investigated the relationship between futile care and moral distress among Iranian nurses and found that moral distress increased with work experience (21). They cited Meltzer and Huckabay, who repeatedly stated that this condition was a product of continuous exposure to moral distress; furthermore, they mentioned aging and mental and physical changes as some other factors that make nurses more vulnerable and more likely to be affected while providing end-of-life care (22). 

**Futile Care and Moral Distress**

The results of this study showed that the perception of futile care in its two dimensions of severity and frequency was at a medium to high level in 72.7% of the ICU nurses. In two separate studies, Piers et al. (28) and Ferrell (29) also found a high level of futile care perception among nurses, which is in accordance with the present study results. These findings are different from those of Rostami et al. (30) and Mohammadi and Roshanzadeh (16). In a 2010 study of the relationship between futile care and moral distress among ICU nurses, Dunwoody and Danielle reported high futile care perception among nurses, which she considered to be a cause for moral distress (31). Mobley et al. conducted a study on the relationship between futile care perception and moral distress in 2007 and reported moderate to high perception levels among nurses; they believed that one reason for moral distress among ICU nurses was greater work experience and higher perception of futile care (15). In the present study, the univariate regression analysis demonstrated a positive correlation between futile care perception and moral distress, which is comparable with the results of the above-mentioned studies. The univariate logistic regression analysis also showed a higher likelihood and occurrence rate of moral distress among male nurses. However, Ebrahimi et al. (32) and other studies (7, 23) discovered no significant relationship between sex and moral distress.

## Conclusion

The results of this study showed that ICU nurses experience a high level of moral distress, which has a positive correlation with ICU work experience as well as futile care perception. Moral distress can lead to low collaboration with doctors, exhaustion and burnout. Therefore, further studies are needed to identify other related factors. Some recommended measures in this respect include training nurses in positive adjustment mechanisms in order to reduce the effects of this phenomenon, and adjusting the ICU staff in a way that nurses with less work experience are used in this ward. One limitation of this research was that although approximately 90% of the ICU nurses of Qom hospitals affiliated to Qom University of Medical Sciences participated in this research, the sample size was 117. 
